# Per- and Polyfluoroalkyl Substances (PFAS) in Michigan: A Novel High-Throughput Method with Liquid Chromatography–Mass Spectrometry (LC-MS/MS) Analysis of 42 PFAS Compounds in Human and Bovine Serum

**DOI:** 10.3390/toxics14030209

**Published:** 2026-02-28

**Authors:** Jessica M. Morrison, Douglas J. Carmack, Sarah Y. Lockwood-O’Brien, Chelsea A. Bielicki, Julia R. Feeley, Robert L. Frisk, Timothy A. Karrer

**Affiliations:** Bureau of Laboratories, State of Michigan Department of Health and Human Services, 3350 North Martin Luther King Junior Boulevard, Lansing, MI 48906, USA; carmackd@michigan.gov (D.J.C.); lockwoodobriens@michigan.gov (S.Y.L.-O.); bielickic@michigan.gov (C.A.B.); feeleyj2@michigan.gov (J.R.F.); friskr@michigan.gov (R.L.F.); karrert@michigan.gov (T.A.K.)

**Keywords:** fluorocarbons, biological monitoring, serum, environmental monitoring, liquid chromatography–mass spectrometry, analytical sample preparation methods

## Abstract

A novel method for the analysis of per- and polyfluoroalkyl substances (PFAS) in serum is presented here, utilizing a matrix-matched calibration curve, small sample size, high-throughput sample prep with a protein crash and filtration, resulting in a concentrated and purified sample, which is analyzed via liquid chromatography (LC) mass spectrometry (MS, LC-MS/MS). As part of the validation of this method, accuracy, precision, reportable range verification, sensitivity, carryover, recovery, specificity, interferences, maximum dilutions, and stability are presented. This method quantitates 42 PFAS compounds across a wide variety of compound classes and has limits of detection in the low ng L^−1^ range with all analytes having a limit of quantitation less than the first calibrator (25 ng L^−1^). Total method accuracy was >91%, exceeding the set performance criteria goal of ≥85%. Method imprecision was <11%, exceeding the set performance criteria goal of ≤15%. Method recovery averaged 85%. Limited and minimal matrix effects were seen. Interference from bile acids was found to be of no concern at biologically relevant concentrations for this method. This method has been successfully employed in biomonitoring studies for residents of Michigan over the past eight years.

## 1. Introduction

In the serene landscapes of Michigan, amidst its sprawling forests and shimmering lakes, lies an unseen threat that has silently infiltrated its ecosystems and communities: per- and polyfluoroalkyl substances (PFAS). These persistent synthetic chemicals, ubiquitous in everyday products [1,2,3,4] and industrial processes [5,6,7,8,9], have emerged as a pressing concern due to their potential association with adverse health effects [6,10,11,12,13,14] and environmental persistence [8,9,15,16]. Among the myriad pathways of exposure, human serum stands as a crucial matrix for assessing PFAS risk and understanding the impact on public health. Herein, we present a pioneering advancement in analytical methodology tailored to confront this looming challenge: a novel approach for the comprehensive analysis of PFAS in human and bovine serum, leveraging the power of high-performance liquid chromatography (LC)–mass spectrometry (MS, LC-MS/MS). Developed against the backdrop of Michigan’s unique environmental landscape and informed by the urgent need for robust analytical techniques, this method demonstrates proven sensitivity, specificity, and high-throughput efficiency in quantifying a diverse array of 42 PFAS compounds.

PFAS are extensively used in consumer and industrial products, including textiles (clothing, carpets, and upholstery) [4], food packaging [17], non-stick cookware [16,18], cosmetics [16,18], and firefighting foams [19] due to their water-, grease-, and heat-resistant properties. Their persistence and widespread application result in continuous human exposure through multiple pathways, from household items to occupational settings. This ubiquity underscores the urgent need for increased awareness, stricter regulatory measures, and the development of truly safer alternatives to mitigate exposure and reduce associated health risks associated with these persistent chemicals.

PFAS have been linked to numerous adverse health effects, including increased risks of kidney, testicular, prostate, and thyroid cancers [6,11,12,13,14]; immune suppression [10,11]; reproductive issues such as reduced fertility and low birth weight [4,6]. Prenatal exposure is associated with developmental delays, altered thyroid hormone levels, and neurodevelopmental disorders [13,15,20]. PFAS can also disrupt endocrine function, affecting metabolism and growth [8,12,16]. These findings highlight the importance of understanding and mitigating exposure to PFAS in various environmental and consumer contexts.

PFAS are highly persistent in the environment due to their strong carbon–fluorine bonds [6], which make them resistant to natural degradation processes such as photolysis, hydrolysis, and microbial metabolism [6,7,15,21,22]. As a result, they can remain in soil, water, and sediment for decades, or even centuries, bioaccumulating in organisms and biomagnifying through food chains [7,8,9,15,21,23,24]. PFAS have been detected globally in surface water, groundwater, soil, air, and biota, with contamination frequently reported near industrial sites, military installations, wastewater treatment plants, and areas where firefighting foams are used [7,8,9,15,21,25,26,27]. Their persistence poses long-term risks to ecosystems and human health, emphasizing the need for comprehensive monitoring, regulatory action, and effective remediation strategies to limit environmental release and mitigate adverse impacts [28,29,30,31,32].

Michigan has a rich history with PFAS and has been at the forefront of PFAS detection for its residents. In 2001, the State of Michigan began testing surface waters for PFAS when 21 streams were sampled for perfluorooctanoic acid (PFOA) and perfluorooctanesulfonic acid (PFOS) contamination [33] following initial United States Environmental Protection Agency (EPA) studies that suggested PFOS can build up in the body [34] and a link was found between liver damage and PFAS exposure [35]. In 2012, PFAS was first discovered in the groundwater at the Wurtsmith Air Force Base in Oscoda Township, Michigan [36], following the development of provisional health advisories for PFOS and PFOA by the EPA [37]. Shortly following, Michigan issued its first “Do Not Eat” fish advisory for PFOS and in waters surrounding the Air Force Base [36] as the first PFAS-related fish advisory for the Michigan “Eat Safe Fish” public guidance document [38]. These advisories have evolved to include PFAS in wild game in addition to fish [39]. Following the lifetime health advisory for PFOA and PFOS issued by the EPA in 2016 [40], the State of Michigan established the Michigan PFAS Action Response Team (MPART) [41,42], issued the first PFAS in foam advisories [43], discovered PFAS in the drinking water of Rockford, Michigan, residents [44], and began the Michigan PFAS Exposure and Health Study in Rockford and Parchment, Michigan [45]. This also resulted in the State of Michigan adopting the EPA lifetime health advisories as environmental cleanup criteria, the development of Michigan Public Health Drinking Water Screening Levels [46], as well as the establishment of maximum contaminant levels (MCLs) for seven types of PFAS [47]. In addition to the method presented here for analysis in serum and the aforementioned fish and tissue analyses, the State of Michigan has also developed methods for the detection of PFAS in water [48] and dried blood spots (DBS). This proven analytical method has been utilized over the past eight years by the State of Michigan Department of Health and Human Services (MDHHS) to quantify 42 PFAS in human serum for statewide and targeted biomonitoring studies, such as the North Kent County Exposure Assessment [44], Michigan Chemical Exposure Monitoring [49], Oscoda Area Exposure Assessment [50], Michigan PFAS Exposure and Health Study [45], PFAS Exposure and Antibody Response to COVID-19 Vaccine Study [51,52], and the PFAS in Firefighters of Michigan Surveillance [41] for almost 8000 Michigan residents.

While other methods are available in the literature for the analysis of PFAS compounds, many do not capture the quantity or breadth of PFAS compounds as studied here, or are capable of quantifying analytes across a broad dynamic range without compromising sensitivity or precision [53,54,55,56,57,58,59,60]. Many methods available in the literature only focus on some of the most common PFAS (perfluorohexanesulfonic acid (PFHxS), PFOA, PFOS) [61,62,63,64,65,66,67,68], whereas the method presented here quantitates 42 PFAS compounds. Of significant note, the extraction method utilized by the Center for Disease Control (CDC) National Health and Nutrition Examination Survey (NHANES) is limited to the analysis of 12 PFAS compounds [69]. Other literature methods are dilute and shoot style methods [61,64,70], utilize on-line solid phase extraction (SPE) [67,69], or utilize traditional SPE [63,65,66,68]. Each of these extraction methods have their own considerations, including higher reporting limits and risks of instrument contamination (dilute and shoot style methods), risks of systemic PFAS contamination and pH considerations (on-line SPE), and potential low recoveries of PFAS analytes of interest (traditional SPE) [53,54,55,71,72]. Our method is a compromise, utilizing a protein crash combined with a small particle size filtration prior to sample analysis which has been found to offer strong recovery of the PFAS compounds of interest for the State of Michigan. In addition to a robust sample extraction, the method as presented here is capable of quantitation of 42 PFAS analytes over a large reportable range (25 ng L^−1^–50 ng mL^−1^). Such a large reportable range is necessary in the State of Michigan to capture low levels as necessary for biomonitoring studies, similar to NHANES [69], as well as direct exposure assessments of residents with significant known PFAS exposure [52,73].

With this method 42 PFAS compounds are analyzed spanning many different classes: perfluorocarboxylic acids (PFBA, PFPeA, PFHxA, PFHpA, PFOA (linear, branched, and total), PFNA, PFDA, PFUnA, PFDoA, PFTriA, PFTeA), perfluorosulfonic acids (PFPrS, PFBS, PFPeS, PFHxS (linear, branched, and total), PFHpS, PFOS (linear, branched, and total), PFNS, PFDS), fluorotelomer sulfonic acids (4:2 FTS, 6:2 FTS, 8:2 FTS), perfluorosulfonamidoacetic acids (MeFOSAA, EtFOSAA), perfluorosulfonamides (PFBSA, PFHxSA, PFOSA), fluorotelomer carboxylic acids (3:3 FTCA, 5:3 FTCA, 7:3 FTCA), and a variety of other new/replacement PFAS (PFECHS, NFDHA, PFEESA, PFMPA, PFMBA, HFPO-DA, ADONA, 9Cl-PF3ONS, 11Cl-PF3OUdS). In addition, the high-throughput nature of the method presented here allows for efficient routine analysis of a reasonable serum sample size (500 µL) while maintaining low ng L^−1^ limits of detection (LOD) that are comparable to the PFAS maximum contaminant levels set by the State of Michigan [41]. In this paper, we delineate the intricacies of our method, elucidating each crucial step from sample preparation to chromatographic separation and mass spectrometric detection. Moreover, we showcase the robustness and reliability of our approach through rigorous validation studies, encompassing method accuracy, precision, sensitivity, linearity, specificity, recovery, and stability.

## 2. Materials and Methods

### 2.1. Chemicals and Reagents

All PFAS compounds included in this method can be found in Table 1 along with their names, abbreviations, and CAS numbers. The materials prepared below each utilize different vendors (Wellington Laboratories (Guelph, ON, Canada), Cambridge Isotope Laboratories (Tewksbury, MA, USA), or Absolute Standards (Hamden, CT, USA)) for their source materials for each analyte in accordance with validation guidelines of the College of the American Pathologists (CAP) and are detailed, along with their available stock concentrations, in Table 1 for simplicity.

An internal standard stock solution was prepared using the labeled internal standard analogs of 20 analytes (^13^C-PFBA, ^13^C-PFPeA, ^13^C-PFHxA, ^13^C-PFHpA, ^13^C-PFOA, ^13^C-PFNA, ^13^C-PFDA, ^13^C-PFUnA, ^13^C-PFDoA, ^13^C-PFTeDA, ^13^C-PFBS, ^13^C-PFHxS, ^13^C-PFOS, ^13^C-4:2FTS, ^13^C-6:2FTS, ^13^C-8:2FTS, ^2^H-N-MeFOSAA, ^2^H-N-EtFOSAA, ^13^C-PFOSA, ^13^C-HFPO-DA) (Table 1) to a concentration of 1.5 ng mL^−1^ in methanol, designed to fall near the mid-point of the calibration curve.

Matrix-matched calibration standards containing all PFAS analytes diluted in Gibco™ charcoal-stripped fetal bovine serum (Thermo Fisher Scientific, Waltham, MA, USA) were prepared in-house at the following concentrations (concentrations listed apply to all analytes unless otherwise noted due to concentration variability in standard materials): 0.025 ng mL^−1^ (0.0203 ng mL^−1^ L-PFHxS, 0.0047 ng mL^−1^ B-PFHxS), 0.05 ng mL^−1^ (0.04055 ng mL^−1^ L-PFHxS, 0.00945 ng mL^−1^ B-PFHxS), 0.1 ng mL^−1^ (0.0811 ng mL^−1^ L-PFHxS, 0.0189 ng mL^−1^ B-PFHxS), 0.5 ng mL^−1^ (0.4055 ng mL^−1^ L-PFHxS, 0.0945 ng mL^−1^ B-PFHxS), 1.0 ng mL^−1^ (0.811 ng mL^−1^ L-PFHxS, 0.189 ng mL^−1^ B-PFHxS), 5.0 ng mL^−1^ (4.055 ng mL^−1^ L-PFHxS, 0.945 ng mL^−1^ B-PFHxS), 10 ng mL^−1^ (8.11 ng mL^−1^ L-PFHxS, 1.89 ng mL^−1^ B-PFHxS), 50 ng mL^−1^ (40.55 ng mL^−1^ L-PFHxS, 9.45 ng mL^−1^ B-PFHxS). Analyte spike solutions were purchased from the vendors at the concentrations as listed in Table 1 for each calibrator material. Gibco™ charcoal-stripped fetal bovine serum was screened for PFAS contamination prior to use. The matrix-matched calibration standards were utilized for calibration curves for all experiments that follow, as well as the test material for the reportable range study (Section 2.4.3), sensitivity and limit of detection study (Section 2.4.4).

Matrix-matched quality control (QC) materials containing all PFAS analytes diluted in Gibco™ charcoal-stripped fetal bovine serum were prepared in-house at the following concentrations (concentrations listed apply to all analytes unless otherwise noted): 0 ng mL^−1^, 0.075 ng mL^−1^ (0.06975 ng mL^−1^ L-PFOS, 0.08025 ng mL^−1^ B-PFOS, 0.0608 ng mL^−1^ L-PFHxS, 0.0142 ng mL^−1^ B-PFHxS), 0.75 ng mL^−1^ (0.6975 ng mL^−1^ L-PFOS, 0.8025 ng mL^−1^ B-PFOS, 0.608 ng mL^−1^ L-PFHxS, 0.142 ng mL^−1^ B-PFHxS), 7.5 ng mL^−1^ (6.975 ng mL^−1^ L-PFOS, 8.025 ng mL^−1^ B-PFOS, 6.08 ng mL^−1^ L-PFHxS, 1.42 ng mL^−1^ B-PFHxS). Analyte spike solutions were purchased from the vendors at the concentrations as listed in Table 1 for each quality control material. Gibco™ charcoal-stripped fetal bovine serum was screened for PFAS contamination prior to use. The matrix-matched quality control materials were utilized for quality control of all experiments that follow, as well as the test material for the ruggedness and stability study (Section 2.4.10).

Matrix-matched laboratory validation samples containing all PFAS analytes diluted in Gibco™ charcoal-stripped fetal bovine serum were prepared in-house at the following concentrations (concentrations listed apply to all analytes unless otherwise noted): 0.0843 ng mL^−1^ (0.169 ng mL^−1^ PFPrS, PFECHS, 3:3 FTCA, 5:3 FTCA, 7:3 FTCA, NFDHA, PFEESA, PFMBA, PFMPA), 0.825 ng mL^−1^ (1.65 ng mL^−1^ PFPrS, PFECHS, 3:3 FTCA, 5:3 FTCA, 7:3 FTCA, NFDHA, PFEESA, PFMBA, PFMPA), 2.75 ng mL^−1^ (1.65 ng mL^−1^ PFPrS, PFECHS, 3:3 FTCA, 5:3 FTCA, 7:3 FTCA, NFDHA, PFEESA, PFMBA, PFMPA). Analytes were spiked using materials from Wellington Laboratories (PFAC-30PAR, PFPrS, PFECHS, FPePA, FPrPA, PHpPA, NFDHA, PFEESA, PFMPA, PFMBA). Charcoal-stripped fetal bovine serum was screened for PFAS contamination prior to use. The matrix-matched laboratory validation samples were utilized as samples for accuracy studies (Section 2.4.1).

Five levels of matrix-matched internal proficiency testing samples containing all PFAS analytes diluted in Gibco™ charcoal-stripped fetal bovine serum were prepared in-house at the following concentrations (concentrations listed apply to all analytes unless otherwise noted): 0 ng mL^−1^, 0.06 ng mL^−1^ (0.0474 ng mL^−1^ L-PFOS, 0.0726 ng mL^−1^ B-PFOS, 0.0588 ng mL^−1^ L-PFOA, 0.0012 ng mL^−1^ B-PFOA, 0.0516 ng mL^−1^ L-PFHxS, 0.0084 ng mL^−1^ B-PFHxS), 0.6 ng mL^−1^ (0.474 ng mL^−1^ L-PFOS, 0.726 ng mL^−1^ B-PFOS, 0.588 ng mL^−1^ L-PFOA, 0.012 ng mL^−1^ B-PFOA, 0.516 ng mL^−1^ L-PFHxS, 0.084 ng mL^−1^ B-PFHxS), 6 ng mL^−1^ (4.74 ng mL^−1^ L-PFOS, 1.26 ng mL^−1^ B-PFOS, 5.88 ng mL^−1^ L-PFOA, 0.12 ng mL^−1^ B-PFOA, 5.16 ng mL^−1^ L-PFHxS, 0.84 ng mL^−1^ B-PFHxS), 24 ng mL^−1^ (18.96 ng mL^−1^ L-PFOS, 5.04 ng mL^−1^ B-PFOS, 23.52 ng mL^−1^ L-PFOA, 0.48 ng mL^−1^ B-PFOA, 20.64 ng mL^−1^ L-PFHxS, 3.36 ng mL^−1^ B-PFHxS). Analytes were spiked using a custom mix from Absolute Standards (2 ug mL^−1^) and P5MHpS (Wellington Laboratories) (0.06 ng mL^−1^, 0.6 ng mL^−1^ samples), or only the custom mix from Absolute Standards (24 ng mL^−1^, 6 ng mL^−1^ samples). Gibco™ charcoal-stripped fetal bovine serum was screened for PFAS contamination prior to use. The matrix-matched internal proficiency testing samples were utilized for precision studies (Section 2.4.2).

The laboratory participates in a proficiency testing (PT) program for PFAS in serum were offered by the Institut National de Santé Publique du Québec (INSPQ), the Arctic Monitoring and Assessment Programme (AMAP). Previously analyzed AMAP PT samples from 2019 (PT round 2) and 2022 (PT round 1) were utilized to assess the accuracy of our method (Section 2.4.1).

The laboratory participates in a PT program for persistent organic pollutants (POPs) in serum, also offered by AMAP. Previously analyzed AMAP specimens for POP analysis were utilized to study POP interferences with PFAS serum analysis by spiking PFAS at the same concentration as the mid-level matrix-matched quality control material, 0.75 ng mL^−1^ (0.6975 ng mL^−1^ L-PFOS, 0.8025 ng mL^−1^ B-PFOS, 0.608 ng mL^−1^ L-PFHxS, 0.142 ng mL^−1^ B-PFHxS). Analyte spike solutions were purchased from the vendors at the concentrations listed in Table 1. These samples were utilized to assess the effect of potential interferences from other common serum contaminants on our method (Section 2.4.8).

National Institute of Standards and Technology (NIST, Gaithersburg, MD, USA) standard reference materials (SRM) were utilized to assess accuracy (standard reference materials 1957 and 1958) in human serum (Section 2.4.1).

An over-range sample containing all PFAS analytes diluted in Gibco™ charcoal-stripped fetal bovine serum was prepared in-house at 500 ng mL^−1^. Analytes were spiked using materials from Wellington Laboratories (PFAC-30PAR, PFPrS, PFECHS, FPePA, FPrPA, PHpPA, NFDHA, PFEESA, PFMPA, PFMBA). This over-range sample was utilized to study carryover for both the instrument and Tomtec Quadra 3 or 4 liquid handlers (Tomtec, Hamden, CT, USA) (Section 2.4.5). Additionally, the over-range sample was utilized to create serial dilutions (1:10, 1:100, 1:1000, 1:10,000) in pre-screened Gibco™ charcoal-stripped fetal bovine serum, as well as individual dilutions (1:10, 1:100, 1:1000, 1:10,000). These dilutions were utilized to understand the maximum dilution possible with the method, as well as the most accurate dilution scheme (Section 2.4.9).

Matrix spikes for method specificity were created at low (0.09 ng mL^−1^) and high (0.9 ng mL^−1^) concentrations, diluted in pre-screened charcoal-stripped fetal bovine serum using materials from Wellington Laboratories (PFAC-24PAR, PFAC-MXF, PFAC-MXG, PFPrS, PFECHS, 3:3 FTCA, 5:3 FTCA, 7:3 FTCA, PFBSA, PFHxSA). Solvent spikes for method specificity were created at low (0.09 ng mL^−1^) and high (0.9 ng mL^−1^) concentrations, diluted in LC-MS grade methanol using materials from Wellington Laboratories (PFAC-24PAR, PFAC-MXF, PFAC-MXG, PFPrS, PFECHS, 3:3 FTCA, 5:3 FTCA, 7:3 FTCA, PFBSA, PFHxSA). These materials together were utilized to study specificity (2.4.6). Solvent spikes for method recovery were created at low (0.45 ng mL^−1^) and high (4.5 ng mL^−1^) concentrations, diluted in LC-MS grade methanol using materials from Wellington Laboratories (PFAC-24PAR, PFAC-MXF, PFAC-MXG, PFPrS, PFECHS, 3:3 FTCA, 5:3 FTCA, 7:3 FTCA, PFBSA, PFHxSA). These materials were utilized to study recovery (Section 2.4.6).

Taurodeoxycholic acid (TDCA, EMD Millipore, Burlington, MA, USA) was prepared at several concentrations including those of biological relevance [74,75] (498.3 ng L^−1^, 4.983 ng mL^−1^, 49.83 ng mL^−1^, 498.3 ng mL^−1^, 4.983 mg L^−1^, 49.83 mg L^−1^, 498.3 mg L^−1^) and mixed with the medium-level (0.75 ng mL^−1^) matrix-matched quality control material to study a potential cross-reactivity with PFAS compounds (Section 2.4.8).

LC-MS grade water, LC-MS grade methanol, LC-MS grade acetonitrile, and LC-MS grade ammonium acetate were purchased from Honeywell Burdick and Jackson (Muskegon, MI, USA).

### 2.2. Sample Preparation

Sample preparation, in brief, consisted of isotopic dilution, protein precipitation, sample filtration, and evaporation/reconstitution which results in a 5x theoretical increase in analyte concentration. Sample preparation utilized a 96-well plate format and was performed using an automated Tomtec Quadra 3 and 4 liquid handler and a 96-well filtration plate (Biotage Isolute Filtration Plate, 20 µm, Uppsala, Sweden). Sample reconstitution was accomplished through mixing via a 96-well plate multi vortexer (VWR, Radnor, PA, USA). Polypropylene TrueTaper 96-well plates were purchased from Analytical Sales and Services (Flanders, NJ, USA).

An aliquot of 60 µL of the internal standard stock solution was added to each well of the 96-well TrueTaper sample plate. Serum samples, calibrators, and quality control materials (500 µL) were added to the appropriate wells of the sample plate. The sample plate was then transferred to the Tomtec (Quadra 3 and Quadra 4), which automatically performed the method which follows. LC-MS grade acetonitrile (1050 µL) was added to each well of the 96-well plate and mixed by aspiration and dispensing nine times. This resulted in protein precipitation. A 96-well filtration plate (Biotage Isolute Filtration Plate, 20 µm) was utilized for sample filtration after the plate was washed and conditioned with LC-MS grade acetonitrile (400 µL) and LC-MS grade water (400 µL). The entire contents of the sample plate were transferred to and filtered through the filter plate into a clean 96-well TrueTaper plate. The eluent was concentrated to dryness under nitrogen using a SPE Dry (Biotage) with a 60 °C upper temperature and an 80 °C lower temperature, with nitrogen flow rate set to 40 L min^−1^. Once the plate dried, it was allowed to cool to room temperature prior to reconstitution with 100 µL LC-MS grade methanol. A 96-well plate vortex mixer was used for sample mixing after a silicone cap mat (Analytical Sales and Services) was placed on top of the 96-well TrueTaper plate (5 min at 1000 rpm).

### 2.3. LC-MS/MS Analysis

The studies that follow in this manuscript were performed on two different instrument systems of which the lab has two of each: Shimadzu Nexera X2 series LC system/Shimadzu 8060 MS (Shimadzu, Kyoto, Japan) and Sciex Exion LC system/Sciex 6500+ MS (Sciex, Framingham, MA, USA). These platforms are all high performance triple quadrupole mass spectrometers in MS/MS multiple reaction monitoring (MRM) negative ion mode using an electrospray ionization (ESI) source. The LC-MS/MS system configuration and data collection were performed using Shimadzu LabSolutions software version 5.97 or Analyst software version 1.7.1 while data processing was performed using LabSolutions Insight software version 3.5 or Sciex OS-MQ software version 2.2. The use of different LC-MS/MS systems from different vendors was not meant to compare the two instrument platforms but was rather based on instrument workload and availability.

Despite the use of two different instrument platforms, LC parameters remained the same between the two platforms. The column ovens consistently maintained a temperature of 40 °C. Two Supelco Ascentis C8 columns (50 mm × 2.1 mm, 3 µm) were utilized, with one column being used as an analytical column and the other being placed between the mixer and the autosampler as a means of delaying PFAS contamination from the LC system. The LC operated with a binary gradient pumping mode utilizing a gradient composed of 2 mM ammonium acetate in LC-MS grade water (mobile phase A, MPA) and LC-MS grade methanol (mobile phase B, MPB) (Table 2). The injection volume was 10 μL.

For the Shimadzu 8060 MS, the mass spectrometer parameters were as follows: nebulizing gas flow 3 L min^−1^, heating gas flow 10 L min^−1^, interface temperature 300 °C, desolvation line temperature 150 °C, heat block temperature 400 °C, drying gas flow 10 L min^−1^. Please see the publication Geiger et al. [48] for the different voltages (quadrupole 1, collision energy, quadrupole 3) for each analyte ion measured.

For the Sciex 6500+ MS, the mass spectrometer parameters were as follows: curtain gas 20 L min^−1^, interface temperature 300 °C, ion source gas 1 30 L min^−1^, ion source gas 2 40 L min^−1^, collision gas 12 L min^−1^, IonSpray voltage −2000 V. See Table 3 for the different voltages (declustering potential, entrance potential, collision energy, and collision exit potential) for each analyte ion measured.

**Figure 1:**
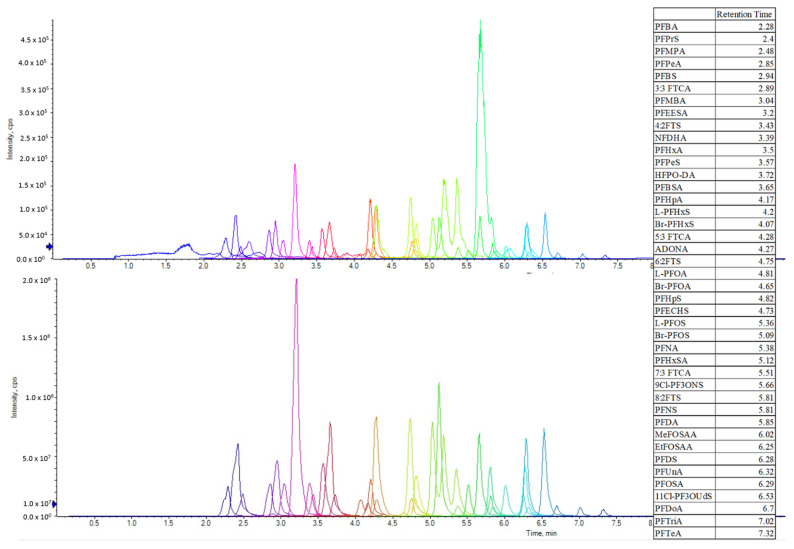
Sciex 6500+ chromatograms: 25 ng L^−1^ (**top**), 50 ng mL^−1^ (**bottom**), with analyte retention times.

In all, these LC and MS parameters result in excellent separation of the 42 PFAS compounds of interest (Figure 1).

### 2.4. Method Validation

For all validation studies that follow, CAP validation guidelines were followed.

#### 2.4.1. Accuracy

Samples used to assess accuracy were NIST-certified reference materials (two samples, 1957 and 1958), previously tested and reported AMAP PT samples (six samples each from 2019 round 2 and 2022 round 1), and laboratory-generated validation samples in bovine serum (three samples). This assessment featured individual samples independently extracted in at least duplicate and ran each day over multiple days by multiple scientists on each of two duplicate LC-MS/MS systems to assess the accuracy of the method. The laboratory’s set performance criteria for accuracy were set to ≥85%. PT sample results were compared to the PT report, NIST samples were evaluated from the material certificate of analysis (COA), and laboratory-generated specimens in bovine serum were evaluated against the calculated expected value. The accuracy for laboratory-generated validation samples was run on two Shimadzu 8060 MS, while the NIST and AMAP accuracy samples were run on two Sciex 6500+ MS.

#### 2.4.2. Precision

The samples used to assess precision were laboratory-generated internal PT materials (five samples). This assessment featured these individual samples being independently extracted in at least duplicate and run each day over multiple days by multiple scientists on each of two duplicate LC-MS/MS systems to assess the inter-day, intra-day, and total precision of the method. The mean and total standard deviation for each precision validator level was calculated and the total coefficient of variation (CV%) was also calculated. Additional variance was assessed for intra-day and inter-day precision. The laboratory’s set performance criteria for total precision was ≤15% imprecision. The precision studies were run on two Sciex 6500+ MS.

#### 2.4.3. Reportable Range

The analytical measurement range was verified by the quantitation of calibration curve points against a calibration curve with R^2^ ≥ 0.9900 to assess accuracy of the analytical measurement range. Throughout the validation study, two calibration curves were individually extracted and ran on each sample plate. For these calculations, each curve was quantitated against the other curve on the same plate. The desired accuracy was set to ±30%, which was the value by which calibration curve point accuracy was assessed at the instrument with each batch run. The reportable range verification was run on two Sciex 6500+ MS.

#### 2.4.4. Sensitivity, Limit of Detection

The limit of detection (LOD) and limit of quantitation (LOQ) was determined by performing a LINEST fit to the calculated concentration for the three lowest calibrators of all calibration curves generated during this validation. The standard deviation in the y-intercept divided by the slope of the least squares fit line equals S0. The analytical LOD was three times S0 and the analytical LOQ was ten times S0. The set performance criteria required that the LOQ must fall below the lowest calibrator, or the assay must be reworked. Data were only used from calibration curves with an R^2^ ≥ 0.9900 and appropriate signal-to-noise ratios (≥10:1 for the quantification ion and ≥3:1 for confirmation ion) with at least ten runs included. Appropriate sensitivity was assessed through signal to noise ratios at the reporting limits of the method. The sensitivity/limit of detection study was performed on both of the two Shimadzu 8060 MS and the two Sciex 6500+ MS.

#### 2.4.5. Carryover

Carryover was assessed for the method for two different scenarios. For instrument carryover, laboratory-generated over-range samples were analyzed followed by a minimum of two subsequent blank injections. Additional carryover from the highest calibrator was also assessed by analyzing blank injections following the end of the calibration curve. This was completed for each instrument used during validation studies: two Shimadzu 8060 MS and two Sciex 6500+ MS.

For carryover occurring during sample preparation, carryover was assessed at the Tomtec liquid handler. The pipette tips were changed following the sample loading step of a laboratory-generated over-range sample and LC-MS grade methanol was transferred using new pipette tips to a clean 96-well plate. This plate was dried down and reconstituted as a sample plate to understand if any residual PFAS was carried over through the Tomtec. Carryover from the laboratory-generated over-range sample on the Tomtec was assessed by looking for peaks in the subsequent plate of blank methanol samples. This study was completed for each Tomtec (Quadra 3 and Quadra 4) utilized for sample preparation.

#### 2.4.6. Recovery

To test method recovery, a known concentration of analyte (low: 0.09 ng mL^−1^; high: 0.90 ng mL^−1^) was processed through the full preparative method, while blank samples were also processed in parallel. At the time of resuspension, the blank sample wells were resuspended with a known concentration in the resuspension solution which should have the same concentration of the spiked/processed well assuming a fully loss-less process. At least ten samples of each condition were tested, and the percent difference between the calculated concentration in bovine serum matrix versus the expected post-spike concentration were used to determine recovery. The average recovery of the low spike and the high spike were used to determine total method recovery.

#### 2.4.7. Specificity

Because this method requires a sample extraction step from the matrix, a test protocol modified from Matuzewki’s “Strategies for the assessment of matrix effect in quantitative bioanalytical methods based on HPLC-MS/MS” was utilized [76]. Standards spiked in solvent at low (0.090 ng mL^−1^) and high concentrations (0.9 ng mL^−1^) were compared to standards spiked in matrix at low (0.90 ng mL^−1^) and high concentrations (0.9 ng mL^−1^) to understand the effect of the matrix on analysis. This study evaluated at least 10 matrix low spikes, 10 matrix high spikes, 10 solvent low spikes, and 10 solvent high spikes, taking the average of each condition. For both the low and the high spike, a ratio (matrix spike:solvent spike) was used to understand signal suppression due to the matrix. An average matrix suppression value above or approaching one indicates no signal suppression due to matrix effects.

#### 2.4.8. Interferences and Cross-Reactivity

To assess additional potential POP interference on PFAS serum analysis, samples were prepared in serum having a high concentration of POP analytes, then analyzed and compared to our matrix-matched quality control materials prepared at the same concentrations. To calculate the interference present from POPs, an average percent difference was calculated.

To assess potential cross-reactivity from bile acids, TDCA was prepared at several concentrations (498.3 ng L^−1^, 4.983 ng mL^−1^, 49.83 ng mL^−1^, 498.3 ng mL^−1^, 4.983 mg L^−1^, 49.83 mg L^−1^, 498.3 mg L^−1^) and mixed with the medium-level matrix-matched quality control material to evaluate the effect of the addition of TDCA on both the chromatography and concentration of the medium-level matrix-matched quality control material (0.75 ng mL^−1^). To calculate the interference present from TDCA, an average percent difference as compared to the medium-level quality control material without TDCA was calculated.

#### 2.4.9. Maximum Dilution

A demonstration of upper-bound range extension has occurred for the method described here to understand the most accurate way to perform dilutions. A 500 ng mL^−1^ spiked matrix sample was prepared and then diluted 10,000× via either (1) a single-step 10,000× dilution, or (2) four iterations of 10× serial dilution. All dilutions occurred in matrix (Gibco™ charcoal-stripped fetal bovine serum). The criteria for acceptability in this case was that the diluted measurements yield ≤15% difference versus the theoretical values after accounting for dilution.

#### 2.4.10. Analyte and/or Matrix Ruggedness (Stability)

To ensure confidence in the ruggedness (stability) of these samples when stored at −20 °C, matrix-matched quality control samples were evaluated over a 20-month period. The criteria for acceptance of sample stability was that quantified values must be maintained at ≤15% difference versus the values observed for freshly prepared samples. An additional test to evaluate ruggedness is freeze–thaw cycling, which simulates the thawing, sampling from, and re-storage of samples stored at ≤−20 °C. Up to five freeze thaw cycles were completed on the matrix-matched quality control materials to evaluate ruggedness.

## 3. Results and Discussion

### 3.1. Accuracy

Accuracy is defined as the degree to which a measure or average of measures is related to a true or reference value. Bias is the quantitative measure of accuracy. Clinical Laboratory Standards Institute (CLSI) document EP15-A2 [77] and International Organization for Standardization (ISO) document 3534-1 [78] define bias as the difference between the expectation of the test result and accepted reference value. Accuracy may also be expressed as trueness. The laboratory’s set acceptance criteria for a passing accuracy result for this method were that all measurements must be within 15% of the expected value.

Three laboratory-generated validation samples were created to assess accuracy and were prepared from materials that originated independently from the calibrator and QC materials. To determine accuracy, the values for all analytes from each level across all batches was assessed and compared with the expected values. The accuracy result for the experimental average of the three samples was within an acceptable ≥91.33% of the target value for all analytes tested (Table 4). The accuracy results of these laboratory-generated samples is enough to consider all analytes fully validated, as the samples analyzed in the paragraphs that follow did not contain all analytes.

In addition to laboratory-created accuracy samples, AMAP PT samples were utilized as a secondary source to measure accuracy. Six individual samples from previously reported AMAP PT events were analyzed. Only analytes which were reported and graded in the AMAP PT report were scored for accuracy. The majority of AMAP sample accuracies hit the desired ≥85% (Table 4, note: some cells are intentionally left blank when that analyte is not present in the sample). However, it is important to note that AMAP PT samples were graded based on an assigned value which is the robust mean of participant results (not a target value), and that there were ranges of acceptance. All the data generated for accuracy for the AMAP samples were assigned a Z-score based on the AMAP PT grading scale to determine if samples would meet the established criteria for the PT. Further investigation shows that all samples analyzed for all analytes would result in an AMAP passing Z-score (set laboratory criteria of Z-score ≤3).

NIST SRM samples were utilized as a third measure of accuracy. Two individual NIST samples (1957 and 1958) were analyzed and compared to the laboratory set performance criteria of an accuracy ≥85%. Only analytes which were listed in the NIST sample certificate of analysis were scored for accuracy (Table 4, note: some cells are intentionally left blank when that analyte is not present in the sample). PFDA and PFUnA, long-chain carboxylic acid PFAS, have accuracies <85%. Due to the way the NIST samples needed to be prepared for this accuracy study, the preparation and storage procedure as recommended by NIST was not completely followed as it was not feasible for this study, and it is anticipated that these differences resulted in lower than acceptable accuracy values. However, PFDA and PFUnA pass accuracy on the two other sample types (laboratory-generated samples and AMAP PT samples); further investigations on these analytes will aid in determining their acceptability for quantitative analysis. Aside from the long-chain PFAS tested, the other analyte accuracies hit the desired ≥85%. It is also important to note that, like the AMAP samples, there were ranges of acceptance for the NIST samples. All the data generated for accuracy for the NIST samples were assigned a pass/fail based on the NIST SRM certificate of analysis acceptable range to determine if samples would pass the accuracy criterion. All of the analytes which had accuracy ≤85% resulted in a failing score, whereas all of the analytes which had accuracy ≥85% resulted in a passing score.

In all, our accuracy measurements suggest superior accuracy as compared to recent similar methods available in the literature, such as Belay et al. (≥72.7%) [53], Da Silva et al. (≥70.07%) [56], and Szabo et al. (≥55%) [72].

### 3.2. Precision

CLSI document EP05-A2 [79] and ISO document 3534-1 [78] define precision as the closeness of agreement between independent test measures under specified conditions [78,79]. This method estimates the total within-laboratory precision for a specific method. Variance associated with factors such as intra-day and inter-day variance were also calculated. For the purposes of this study, all precision results were reported as imprecision. The desired imprecision should be ≤15%. To determine precision, each laboratory-generated internal PT sample was individually extracted at least in duplicate within a run (intra-day precision) over at least five days (inter-day precision). All analytes had <11% imprecision when analyzed for both intra-day and inter-day precision (Table 4).

Our precision measurements suggest comparable precision as Belay et al. listed a precision range of 1.3–11.0% [53] and Lindstrom et al. reported 12–15% [80] whereas the range of our imprecision was 3.05–10.52%.

### 3.3. Reportable Range

Analytical measurement range accuracy was verified by quantitation of individual unique calibration curve points against a passing calibration curve to assess accuracy over the analytical measurement range. The accuracy of each calibration point was averaged to obtain an accuracy across the reportable range. The desired accuracy was set to ±30%, which is the value by which calibration curve point accuracy is assessed at the instrument with each batch run. Reportable range accuracy for each analyte meets a range of 98.29–104.53% showing great accuracy across the reportable range (Table 5).

### 3.4. Sensitivity, Limit of Detection

The LINEST fit was utilized to determine the LOD and LOQ. Data were only utilized from calibration curves with R^2^ ≥ 0.9900 and appropriate signal-to-noise ratios (≥10:1 for the quantification ion and ≥3:1 for confirmation ion) and amounted to up to 84 individual batches per instrument platform. The desired result was LOQ ≤ lowest calibrator (S1) with the goal of using S1 as the reporting limit for the assay. For each analyte, the LOQ ≤ S1 (Table 5). Therefore, S1 was utilized as the reporting limit for the assay (25 ng L^−1^ for all analytes, except L-PFHxS (20 ng L^−1^) and B-PFHxS (5 ng L^−1^). Note: values shown in Table 5 were from the sensitivity study on the Sciex 6500+ MS; however, the similar study on the Shimadzu 8060 MS also resulted in all LOQ ≤ S1.

Our sensitivity data suggest superior detection at low levels as compared to the limits of quantification reported by Belay et al. (8.9–27 ng L^−1^) [53] and Kaiser et al. (12–2800 ng L^−1^) [71] whereas the limits of quantification of this method have a much lower range (3.6–24.4 ng L^−1^) for the same compounds.

### 3.5. Carryover

Instrument carryover characterizes the level of carryover occurring after high or over-range samples are run. With this information, it can be understood whether subsequent measurements following high or over-range samples can be trusted as accurate. Carryover at the Tomtec liquid handlers (Quadra 3 and Quadra 4) was evaluated for each Tomtec utilized during the studies presented here. No carryover was documented in the solvent pipetted after the Tomtec transferred high concentration materials. Carryover at both the Sciex 6500+ MS and Shimadzu 8060 MS systems were evaluated by analyzing blank injections following the highest calibrator as well as over-range samples. No analyte peaks were observed in the blank injections following either the high calibrator or the over-range sample for either instrument platform.

### 3.6. Recovery

A preparative method’s recovery is an indication of the degree to which the analyte material is conserved through processing. The recovery of this method was between 54.13 and 129.57% (Table 6), which is similar to that reported by Kaiser et al. [71] and Szabo et al. [72]. The fluorotelomer sulfonate, 6:2 FTS, had a recovery of ~130%; however, 6:2 FTS had been observed in background levels in the blank serum used for the spike/recovery study, which had a profound effect on the low sample concentration and skewed results. The median of the analyte recoveries was 85.24%, which showed exceptional recovery from the matrix and throughout the method. The majority of analytes with the lowest recoveries (PFBA-72.96%, PFTriA-78.03%, PFTeA-76.57%, PFNS-79.96%, PFDS-73.63%, PFHxSA-76.39%, 3:3FTCA-71.21%, 5:3FTCA-78.43%, 7:3FTCA-54.13%, and 9Cl-PF30NS-67.56%) do not have matching labeled internal standards, which could be a contributing factor to the low recoveries.

### 3.7. Specificity

A test protocol modified from Matuzewki’s “Strategies for the assessment of matrix effect in quantitative bioanalytical methods based on HPLC-MS/MS” [76] was utilized to assess specificity, or the signal suppression due to matrix effects (the degree of matrix suppression observed for samples prepared in matrix relative to samples prepared in pure solvent). An appreciable quantitative difference should not occur for a method using response normalization via stable-labeled internal standards when the matrix does not contribute false signal [76]. At both low and high concentrations throughout the reportable range, a ratio (matrix spike:solvent spike) was used to understand signal suppression due to matrix. According to Matuzewski, an average matrix suppression value above or approaching one indicates no signal suppression due to matrix effects [76]. The majority of PFAS compounds show results around a value of one, which signifies no signal suppression due to matrix effects (Table 6). Several compounds experience values significantly greater than one, including 6:2 FTS (1.64), PFBSA (3.14), and PFHxSA (2.86). Additionally, 7:3 FTCA experienced a value much less than one (0.70). The fluorotelomer sulfonic acid, 6:2 FTS, is an analyte which is frequently found as a contaminant in PFAS studies and in the blank serum used for the study, which could contribute to its matrix enhancement. It was possible that PFBSA and PFHxSA, as well as 7:3 FTCA, experienced matrix issues due to the lack of a matching internal standard.

Every effort was made during the development of this analytical method to avoid inaccuracy due to the interfering signal (e.g., endogenous or environmental chemical interferents, degradation products that occurred during handling or storage, impurities in internal standards, or isomers present in the sample). This LC-MS/MS method facilitates specificity in multiple ways, including: (1) through preparative sample cleanup via protein precipitation and filtration, which can remove chemical interferents; (2) through LC separations including the use of a delay column, which can resolve chemical interferents away from analytes; (3) and through MS, which can eliminate co-occurring chemical interference by permitting signals only from selective ion fragmentation pathways. Additionally, with the use of isotopically labeled internal standards, matrix effects can also be minimized. However, depending on the available isotopically labeled internal standards, a good fit may not be able to be made to prevent matrix suppression.

### 3.8. Interferences and Cross-Reactivity

To establish further proof of matrix specificity, beyond what was discussed in the section above, a more in-depth study of potential interferences and cross-reactivity was performed. Quality control samples in serum matrix were assessed for accuracy versus the same QC samples mixed with high lipid samples with high concentrations of persistent organic pollutants, with the idea being that interference from a large amount of other non-PFAS analytes originating in the serum matrix may result in different results between the two sample types. In general, there was <3% difference between QCs in serum versus QCs mixed with the AMAP POP samples. Interferences from other POPs do not interfere with PFAS quantitation (Table 6).

Additionally, bile acids have been suggested to interfere with PFAS serum analysis of some analytes [75]. TDCA is a bile acid which can be present in human serum and may cause chromatographic interferences for certain PFAS compounds (specifically PFOS, which is ion-related, and PFNA, which is retention time related) [75]. At concentrations ≤498.3 ng mL^−1^ TDCA, no effect was seen on the chromatography or PFAS concentration (Table 7). However, at concentrations ≥4.983 mg L^−1^ TDCA, chromatography suffers and linear and branched PFOS can no longer be distinguished; PFNA was not affected at this concentration. At concentrations ≥498.3 mg L^−1^ TDCA, PFOS and PFNA and their isotopically labeled internal standards were no longer able to be quantitated due to extreme background from the TDCA. Considering biological relevance, though, our method should not be concerned about interference from TDCA. A paper from Blaschka et al. suggests bovine TDCA levels are not higher than 486 ng mL^−1^ at any point in their life cycle [74] and a similar study by Yeung et al. suggested the highest TDCA result quantitated was 772 ng L^−1^ (chicken blood) [75]. A reference for TDCA levels in human samples could not be found. With that literature knowledge, the concentrations in which the effects from TDCA were seen are not of biological relevance and are extreme conditions that may never be seen in reality. It is important to note that once extreme TDCA effects were seen, chromatography suffered to the point that integration was not possible, so it is highly unlikely data would be reported using this method with TDCA effects.

### 3.9. Maximum Dilution

The reportable range may extend beyond the upper bound of the analytical range if over-range samples can be accurately diluted to fit within the analytical range using a matrix-matched diluent. A demonstration of upper-bound range extension has occurred for the method described here to investigate whether a single-step 10,000× dilution, or four iterations of 10× serial dilution are more accurate and therefore which was the preferred dilution scheme for this method. The criteria for acceptability in this case was that the diluted measurements yield ≤ 15% difference versus the theoretical values after accounting for dilution. The data indicates that four iterations of 10× serial dilution to make an overall 10,000× dilution yields quantitative results that were within 11% of the expected value (Table 8). In comparison, a 10,000× single-step dilution was demonstrated to yield results within 14% of the expected value. Both the single-step dilution and the serial dilution meet the set performance criteria for the method; however, due to limitations in the amount of clean diluent that can be reasonably used, serial dilutions were preferred. With the demonstrated utility of 10,000× dilution by 10× serial dilution and a 10,000× dilution by single-step dilution, the reportable range of this method was 0.025–100,000 ng mL^−1^ for all analytes.

### 3.10. Analyte and/or Matrix Ruggedness (Stability)

Samples submitted for PFAS serum analysis are to be maintained at ≤−20 °C for long-term storage. To ensure confidence in the ruggedness (stability) of these samples when stored at ≤−20 °C, matrix-spiked QC samples were evaluated over a 20-month period. The criteria for acceptance of sample stability was that quantified QC values must be maintained at ≤15% difference versus the values observed for freshly prepared QC samples. The results for long-term spiked matrix stability at −20 °C, determined by comparing freshly prepared versus aged QC samples, indicated that all compounds had degraded by ≤11.8% (Table 9, note: some cells are intentionally left blank when that analyte was not present in the sample). This study was completed using materials which only included 24 analytes and further long-term stability studies will be performed with materials containing all 42 analytes.

An additional test to evaluate ruggedness is freeze–thaw cycling, which simulates the thawing, sampling from, and re-storage of samples stored at −20 °C. It is ideal to have the capability of sampling from a specimen multiple times, which may be necessary in cases of analytical failures or sample re-preparation if the specimen’s values or that of a preceding sample are over the analytical range. The criteria for acceptability for freeze–thaw cycling was to demonstrate that the quantified values for QC samples from freeze–thaw cycles 2 through 5 exhibit ≤ 15% degradation relative to the QC samples from freeze–thaw cycle 1. The result for spiked matrix ruggedness to freeze–thaw cycling was that all analytes for freeze–thaw cycles 2 through 5 exhibited ≤10.8% degradation versus the values for freeze–thaw cycle 1, indicating an acceptable condition (Table 9, note: some cells are intentionally left blank when that analyte was not present in the sample).

## 4. Conclusions

This manuscript presented a novel method for the LC-MS/MS analysis of 42 PFAS analytes in a serum matrix, which features a matrix-matched calibration curve, a reasonably low sample volume, and a simple sample preparation with analyte concentration to produce LODs in the low ng L^−1^ range. This method meets and exceeds the desired requirements of accuracy, precision, specificity, carryover, robustness, sensitivity, and reportable range verification. For the past eight years, this established method has been used to report biomonitoring data for Michigan’s residents, and it holds immense potential for enhancing our understanding of PFAS exposure profiles in Michigan’s population and beyond, thereby facilitating targeted regulatory measures and public health interventions. As we continue to refine and apply this methodology in epidemiological studies and environmental monitoring efforts, we envision a future where informed decision-making and proactive measures mitigate the adverse effects of PFAS contamination on human health and the environment.

The views expressed in this article are those of the authors and do not necessarily represent the view or policies of the Michigan Department of Health and Human Services.

The Michigan Department of Health and Human Services (MDHHS) does not discriminate against any individual or group on the basis of race, national origin, color, sex, disability, religion, age, height, weight, familial status, partisan considerations, or genetic information. Sex-based discrimination includes, but is not limited to, discrimination based on sexual orientation, gender identity, gender expression, sex characteristics, and pregnancy.

## Figures and Tables

**Table 1:** PFAS analyte material sources with associated abbreviations, CAS number, source and concentration of the stock utilized for the calibrator material and QC materials, and whether a matching internal standard was utilized (“X”).

Abbreviated Name	Name	CAS#	Calibrator Material	QC Material	ISTD (WL)
PFBA	Perfluorobutanoic acid	375-22-4	WL, 50 mg L^−1^	CIL, 50 mg L^−1^	X
PFPeA	Perfluoropentanoic acid	2706-90-3	WL, 50 mg L^−1^	WL, 50 mg L^−1^	X
PFHxA	Perfluorohexanoic acid	307-24-4	WL, 50 mg L^−1^	Abs, 1000 mg L^−1^	X
PFHpA	Perfluoroheptanoic acid	375-85-9	WL, 50 mg L^−1^	CIL, 50 mg L^−1^	X
L-PFOA	Perfluorooctanoic acid	335-67-1	WL, 50 mg L^−1^	CIL, 50 mg L^−1^	X
B-PFOA	Perfluoro-5-methylheptanoic acid Perfluoro-5,5-dimethylhexanoic acid	335-67-1	WL, 2 mg L^−1^	WL, 2 mg L^−1^	
PFNA	Perfluorononanoic acid	375-95-1	WL, 50 mg L^−1^	CIL, 50 mg L^−1^	X
PFDA	Perfluorodecanoic acid	335-76-2	WL, 50 mg L^−1^	Abs, 1000 mg L^−1^	X
PFUnA	Perfluoroundecanoic acid	2058-94-8	WL, 50 mg L^−1^	WL, 50 mg L^−1^	X
PFDoA	Perfluorododecanoic acid	307-55-1	WL, 50 mg L^−1^	WL, 50 mg L^−1^	X
PFTriA	Perfluorotridecanoic acid	72629-94-8	WL, 50 mg L^−1^	CIL, 50 mg L^−1^	
PFTeA	Perfluorotetradecanoic acid	376-06-7	WL, 50 mg L^−1^	CIL, 50 mg L^−1^	X
PFPrS	Sodium Perfluoropropanesulfonic acid	423-41-6	WL, 50 mg L^−1^	WL, 50 mg L^−1^	
PFBS	Perfluorobutanesulfonic acid	375-73-5	WL, 50 mg L^−1^	Abs, 50 mg L^−1^	X
PFPeS	Perfluoropentanesulfonic acid	2706-91-4	WL, 50 mg L^−1^	Abs, 1000 mg L^−1^	
L-PFHxS	Perfluorohexanesulfonic acid (linear)	355-46-4	WL, 40.55 mg L^−1^	WL, 40.55 mg L^−1^	X
B-PFHxS	Perfluorohexanesulfonic acid (branched)	355-46-4	WL, 9.45 mg L^−1^	WL, 9.45 mg L^−1^	
PFHpS	Perfluoroheptanesulfonic acid	375-92-8	WL, 50 mg L^−1^	WL, 50 mg L^−1^	
L-PFOS	Perfluorooctanesulfonic acid	1763-23-1	WL, 50 mg L^−1^	CIL, 46.5 mg L^−1^	X
B-PFOS	Sodium perfluoro-5-methylheptanesulfonate Sodium perfluoro-5,5-dimethylhexanesulfonate	1763-23-1	WL, 1 mg L^−1^	CIL, 3.5 mg L^−1^	
PFNS	Perfluorononanesulfonic acid	68259-12-1	WL, 50 mg L^−1^	WL, 50 mg L^−1^	
PFDS	Perfluorodecanesulfonic acid	335-77-3	WL, 50 mg L^−1^	WL, 50 mg L^−1^	
4:2 FTS	1H, 1H, 2H, 2H, perfluorohexane sulfonic acid	757124-72-4	WL, 50 mg L^−1^	Abs, 50 mg L^−1^	X
6:2 FTS	1H, 1H, 2H, 2H, perfluorooctane sulfonic acid	27619-97-2	WL, 50 mg L^−1^	Abs, 50 mg L^−1^	X
8:2 FTS	1H, 1H, 2H, 2H, perfluorodecane sulfonic acid	39108-34-4	WL, 50 mg L^−1^	WL, 50 mg L^−1^	X
MeFOSAA	N-Methylperfluorooctane sulfonamidoacetic acid	2355-31-9	WL, 50 mg L^−1^	CIL, 50 mg L^−1^	X
EtFOSAA	N-Ethylperfluorooctane sulfonamidoacetic acid	2991-50-6	WL, 50 mg L^−1^	CIL, 50 mg L^−1^	X
PFBSA	Perfluorobutanesulfonamide	30334-69-1	WL, 50 mg L^−1^	CIL, 50 mg L^−1^	
PFHxSA	Perfluorohexanesulfonamide	41997-13-1	WL, 50 mg L^−1^	CIL, 50 mg L^−1^	
PFOSA	Perfluorooctanesulfonamide	754-91-6	WL, 50 mg L^−1^	WL, 50 mg L^−1^	X
3:3 FTCA	2H,2H,3H,3H-perfluorohexanoic acid	356-02-5	WL, 50 mg L^−1^	Abs, 1000 mg L^−1^	
5:3 FTCA	2H,2H,3H,3H-Perfluorooctanoic acid	914637-49-3	WL, 50 mg L^−1^	Abs, 1000 mg L^−1^	
7:3 FTCA	2H,2H,3H,3H-Perfluorodecanoic acid	812-70-4	WL, 50 mg L^−1^	Abs, 1000 mg L^−1^	
PFecHS	Potassium Perfluoro-4-ethylcyclohexanesulfonate	67584-42-3	WL, 50 mg L^−1^	WL, 50 mg L^−1^	
NFDHA	Nonafluoro-3,6-dioxaheptanoic acid	151772-58-6	WL, 50 mg L^−1^	CIL, 100 mg L^−1^	
PFEESA	Perfluoro (2-ethoxyethane) sulfonic acid	113507-82-7	WL, 50 mg L^−1^	CIL, 50 mg L^−1^	
PFMPA	Perfluoro-3-methoxypropanoic acid	377-73-1	WL, 50 mg L^−1^	Abs, 50 mg L^−1^	
PFMBA	Perfluoro-4-methoxybutanoic acid	863090-89-5	WL, 50 mg L^−1^	Abs, 50 mg L^−1^	
HFPO-DA	Hexafluoropropylene oxide dimer acid	13252-13-6	CIL, 100 mg L^−1^	CIL, 100 mg L^−1^	X
ADONA	Dodecafluoro-3H-4,8-dioxanonanoate	919005-14-4	WL, 50 mg L^−1^	WL, 50 mg L^−1^	
9Cl-PF3ONS	9-chlorohexadecafluoro-3-oxanonane-1-sulfonate	756426-58-1	WL, 50 mg L^−1^	WL, 50 mg L^−1^	
11Cl-PF3OUdS	11-chloroeicosafluoro-3-oxaundecane-1-sulfonate	763051-92-9	WL, 50 mg L^−1^	WL, 50 mg L^−1^	

WL (Wellington Laboratories), CIL (Cambridge Isotope Laboratories), Abs (Absolute Standards).

**Table 2:** LC parameters.

Time (min.)	Flow (mL/min)	MPA Conc (%)	MPB Conc (%)
0.00	0.3000	95.0	5.0
2.00	0.3000	95.0	5.0
8.00	0.3000	50.0	50.0
13.00	0.3000	15.0	85.0
14.00	0.3000	0.0	100.0
14.01	0.3000	95.0	5.0
15.00	0.3000	95.0	5.0

**Table 3:** Sciex 6500+ MS operational parameters. Note: parameters listed for PFOA, PFHxS, and PFOS apply to both linear and branched isomers.

Ion	Precursor (*m*/*z*)	Product (*m*/*z*)	DP (V)	EP (V)	CE (V)	CXP (V)
PFBA	213.0	169.0	−10	−2	−15	−5
PFBA_IS	217.00	172.00	−10	−2	−15	−5
PFPeA	263.00	219.00	−15	−7	−15	−7
PFPeA_C	263.00	263.00	−6	−8	−8	−5
PFPeA_IS	268.00	223.10	−17	−7	−12	−7
PFHxA	313.00	119.000	−17	−12	−25	−7
PFHxA_C	313.00	269.000	−5	−10	−15	−5
PFHxA_IS	318.00	273.00	−15	−10	−15	−5
PFHpA	363.00	169.00	−5	−7	−25	−15
PFHpA_C	363.00	319.00	−12	−7	−15	−10
PFHpA_IS	367.00	322.00	−12	−7	−15	−17
PFOA	413.00	369.00	−20	−2	−15	−10
PFOA_C	413.00	169.00	−20	−2	−27	−10
PFOA_IS	421.00	376.00	−22	−5	−15	−7
PFNA	463.00	169.00	−12	−10	−27	−5
PFNA_C	463.00	419.00	−10	−2	−17	−12
PFNA_IS	472.00	427.00	−7	−2	−17	−12
PFDA	513.00	269.00	−20	−5	−27	−17
PFDA_C	513.00	469.00	−35	−5	−17	−20
PFDA_IS	519.00	474.00	−37	−5	−17	−17
PFUnA	563.00	269.00	−30	−2	−27	−5
PFUnA_C	563.00	519.00	−32	−2	−17	−10
PFUnA_IS	570.00	525.00	−25	−15	−27	−10
PFDoA	613.00	319.00	−30	−2	−27	−10
PFDoA_C	613.00	569.00	−25	−2	−20	−10
PFDoA_IS	615.00	570.00	−22	−12	−17	−20
PFTriA	663.00	319.00	−25	−7	−32	−17
PFTriA_C	663.00	619.00	−30	−2	−20	−15
PFTeA	713.00	319.00	−20	−10	−32	−17
PFTeA_C	713.00	669.00	−20	−12	−22	−7
PFTeA_IS	715.00	670.00	−20	−7	−22	−7
PFBS	299.00	80.00	−72	−7	−60	−12
PFBS_C	299.00	99.00	−75	−7	−42	−5
PFBS_IS	302.00	99.00	−72	−7	−40	−5
PFPeS	349.00	80.00	−80	−12	−67	−7
PFPeS_C	349.00	99.00	−60	−2	−45	−5
PFHxS	399.00	80.00	−67	−7	−60	−10
PFHxS_C	399.00	99.00	−67	−7	−60	−10
PFHxS_IS	402.00	79.85	−65	−5	−52	−5
PFHpS	449.00	80.00	−85	−2	−90	−7
PFHpS_C	449.00	99.00	−85	−12	−80	−5
PFOS	499.00	80.00	−35	−2	−77	−9
PFOS_C	499.00	99.00	−40	−2	−75	−12
PFOS_IS	507.00	99.00	−35	−7	−72	−5
PFNS	549.00	80.00	−52	−7	−110	−12
PFNS_C	549.00	99.00	−45	−2	−90	−5
PFDS	599.00	80.00	−85	−2	−117	−12
PFDS_C	599.00	99.00	−82	−2	−92	−12
PFOSA	498.00	78.00	−95	−2	−97	−12
PFOSA_IS	506.00	78.00	−92	−2	−92	−12
4:2FTS	327.00	307.00	−37	−2	−30	−10
4:2FTS_C	327.00	81.00	−37	−5	−43	−5
4:2FTS_IS	329.00	81.00	−32	−2	−39	−10
6:2FTS	427.00	407.00	−52	−12	−35	−12
6:2FTS_C	427.00	81.00	−67	−2	−55	−10
6:2FTS_IS	429.00	81.00	−67	−2	−57	−12
8:2FTS	527.00	507.00	−75	−5	−40	−10
8:2FTS_C	527.00	81.00	−72	−2	−75	−10
8:2FTS_IS	529.00	81.00	−65	−2	−77	−7
MeFOSAA	570.00	419.00	−50	−2	−30	−12
MeFOSAA_C	570.00	169.00	−50	−5	−40	−5
MeFOSAA_IS	573.00	419.00	−50	−5	−30	−12
EtFOSAA	584.00	419.00	−52	−5	−30	−12
EtFOSAA_C	584.00	526.00	−52	−10	−32	−5
EtFOSAA_IS	589.00	419.00	−52	−12	−30	−20
PFPrS	249.00	79.85	−40	−7	−50	−9
PFPrS_C	249.00	99.10	−35	−7	−40	−9
PFMPA	229.10	84.95	−5	−5	−30	−8
PFMBA	279.00	84.90	−4	−7	−33	−8
PFEESA	315.00	134.85	−15	−6	−35	−6
PFEESA_C	315.00	69.05	−30	−6	−75	−6
NFDHA	201.20	84.90	−20	−5	−25	−9
HFPO-DA	285.00	169.00	−17	−7	−15	−5
HFPO-DA_C	285.00	185.00	−17	−5	−25	−10
HFPO-DA_IS	287.00	169.00	−17	−5	−12	−5
PFBSA	298.10	78.05	−45	−8	−45	−9
PFBSA_C	298.10	48.10	−45	−7	−95	−5
ADONA	377.00	251.00	−15	−10	−20	−15
ADONA_C	377.00	85.00	−12	−7	−37	−10
PFECHS	461.00	381.10	−55	−8	−40	−7
PFECHS_C	461.00	99.05	−55	−8	−55	−8
PFHxSA	398.00	77.85	−40	−9	−75	−9
PFHxSA_C	398.00	169.20	−75	−9	−35	−9
9Cl-PF3ONS	531.00	351.00	−17	−2	−37	−20
9Cl-PF3ONS_C	531.00	82.90	−20	−2	−35	−12
11Cl-PF3OUdS	631.0	451.0	−42	−2	−45	−17
11Cl-PF3OUdS_C	631.0	82.90	−45	−2	−60	−10
3:3FTCA	241.1	177.1	−8	−6	−10	−5
3:3FTCA_C	241.1	117.15	−9	−8	−50	−10
5:3FTCA	341.1	237.15	−25	−10	−20	−10
5:3FTCA_C	341.1	217.15	−25	−10	−35	−10
7:3FTCA	441.1	317.15	−30	−7	−6	−6
7:3FTCA_C	441.1	337.05	−25	−7	−6	−6

**Table 4:** Accuracy and precision. L—linear, B—branched, T—total. Some cells are intentionally left blank (grey cell) when that analyte is not present in the sample.

	Imprecision, Inter-Day	Imprecision, Intra-Day	Accuracy, (Lab Samples)	Accuracy, AMAP	Z-Scores, AMAP	Accuracy, NIST
PFBA	6.60%	4.61%	91.33%	96.79%	−0.08	
PFPeA	5.51%	3.74%	94.07%			
PFHxA	5.31%	3.64%	97.39%	93.19%	−0.08	
PFHpA	5.56%	3.62%	96.22%	88.46%	0.66	88.45% (PASS)
L-PFOA	6.86%	4.09%	97.71%	86.42%	0.31	95.92% (PASS)
B-PFOA	4.19%	3.05%	100.00%			
PFNA	5.93%	4.36%	96.77%	87.29%	0.79	93.24% (PASS)
PFDA	6.67%	5.13%	98.10%	85.95%	0.43	59.56% (FAIL)
PFUnA	7.31%	4.58%	98.82%	88.75%	0.54	68.45% (FAIL)
PFDoA	7.71%	5.64%	98.62%			
PFTriA	10.52%	7.26%	96.20%			
PFTeA	5.25%	3.26%	98.37%			
PFPrS	7.30%	4.76%	95.60%			
PFBS	5.15%	3.27%	95.95%	85.33%	−0.06	
PFPeS	6.98%	4.81%	96.25%			
L-PFHxS	5.77%	3.95%	92.89%	82.57%	0.97	90.22% (PASS)
B-PFHxS	9.36%	6.48%	93.35%			
PFHpS	6.15%	4.23%	94.95%	70.17%	2.17	
L-PFOS	8.69%	5.56%	94.91%	70.31% (L) 81.87% (B) 87.13% (T)	1.90 (L) −0.80 (B) 0.65 (T)	93.67% (T) (PASS)
B-PFOS	9.89%	6.13%	96.71%			
PFNS	8.15%	5.56%	96.05%			
PFDS	8.04%	5.42%	94.56%			
4:2 FTS	7.02%	5.20%	96.56%			
6:2 FTS	7.34%	5.13%	96.76%			
8:2 FTS	8.25%	4.91%	97.35%			
MeFOSAA	5.99%	4.36%	97.21%			
EtFOSAA	6.43%	4.35%	96.22%			
PFBSA	8.70%	5.84%	95.19%			
PFHxSA	8.21%	6.05%	92.47%			
PFOSA	5.66%	3.82%	96.56%			
3:3 FTCA	10.05%	7.07%	92.33%			
5:3 FTCA	8.11%	5.79%	94.05%			
7:3 FTCA	9.15%	5.37%	95.66%			
PFECHS	6.28%	4.72%	92.25%			
NFDHA	5.51%	3.94%	93.36%			
PFEESA	7.15%	4.34%	93.01%			
PFMPA	6.12%	3.85%	95.87%			
PFMBA	7.52%	5.27%	94.37%			
HFPO-DA	5.90%	3.75%	98.96%			
ADONA	5.90%	3.68%	94.62%			
9Cl-PF3ONS	6.95%	4.97%	96.07%			
11Cl-PF3OUdS	10.05%	6.97%	91.73%			

**Table 5:** Sensitivity (LOD/LOQ) and reportable range accuracy. All values are reported in ng mL^−1^.

	SD/m (S0)	LOD (3 × S0)	LOQ (10 × S0)	S1	Reportable Range Accuracy
PFBA	0.0023	0.0068	0.0227	0.025	103.13%
PFPeA	0.0013	0.0039	0.0130	0.025	100.00%
PFHxA	0.0010	0.0030	0.0101	0.025	101.56%
PFHpA	0.0011	0.0033	0.0110	0.025	100.00%
L-PFOA	0.0021	0.0064	0.0214	0.025	100.00%
B-PFOA	0.0024	0.0071	0.0234	0.025	100.74%
PFNA	0.0018	0.0053	0.0178	0.025	100.00%
PFDA	0.0021	0.0064	0.0215	0.025	101.56%
PFUnA	0.0018	0.0054	0.0181	0.025	100.00%
PFDoA	0.0013	0.0040	0.0134	0.025	101.84%
PFTriA	0.0020	0.0061	0.0202	0.025	101.88%
PFTeA	0.0017	0.0051	0.0171	0.025	101.79%
PFPrS	0.0009	0.0027	0.0089	0.025	103.13%
PFBS	0.0008	0.0024	0.0080	0.025	98.44%
PFPeS	0.0021	0.0064	0.0212	0.025	100.47%
L-PFHxS	0.0014	0.0041	0.0136	0.02	101.56%
B-PFHxS	0.0004	0.0011	0.0036	0.005	99.99%
PFHpS	0.0016	0.0047	0.0158	0.025	100.63%
L-PFOS	0.0021	0.0064	0.0215	0.025	103.13%
B-PFOS	0.0012	0.0035	0.0117	0.025	101.56%
PFNS	0.0024	0.0073	0.0244	0.025	103.81%
PFDS	0.0016	0.0049	0.0163	0.025	104.53%
4:2 FTS	0.0024	0.0072	0.0241	0.025	101.25%
6:2 FTS	0.0023	0.0070	0.0234	0.025	103.27%
8:2 FTS	0.0013	0.0038	0.0126	0.025	102.18%
MeFOSAA	0.0010	0.0031	0.0104	0.025	101.79%
EtFOSAA	0.0015	0.0045	0.0150	0.025	102.70%
PFBSA	0.0013	0.0040	0.0133	0.025	99.40%
PFHxSA	0.0012	0.0037	0.0123	0.025	99.90%
PFOSA	0.0022	0.0065	0.0217	0.025	103.13%
3:3 FTCA	0.0022	0.0066	0.0219	0.025	99.78%
5:3 FTCA	0.0021	0.0063	0.0209	0.025	98.29%
7:3 FTCA	0.0016	0.0049	0.0163	0.025	99.55%
PFECHS	0.0010	0.0031	0.0104	0.025	101.56%
NFDHA	0.0023	0.0068	0.0226	0.025	98.59%
PFEESA	0.0018	0.0055	0.0184	0.025	100.00%
PFMPA	0.0009	0.0026	0.0088	0.025	100.00%
PFMBA	0.0013	0.0039	0.0128	0.025	99.12%
HFPO-DA	0.0024	0.0071	0.0236	0.025	101.46%
ADONA	0.0013	0.0038	0.0126	0.025	101.25%
9Cl-PF3ONS	0.0018	0.0053	0.0175	0.025	100.00%
11Cl-PF3OUdS	0.0015	0.0045	0.0149	0.025	102.36%

**Table 6:** Method recovery, specificity, and POP interferences.

	% Recovery	Specificity	% Recovery from POP Serum
PFBA	72.96%	0.92	101.44%
PFPeA	86.49%	1.06	98.06%
PFHxA	94.19%	1.14	100.91%
PFHpA	91.01%	1.02	98.84%
L-PFOA	85.72%	1.01	99.48%
B-PFOA	100.00%	1.00	100.00%
PFNA	83.68%	0.95	100.99%
PFDA	84.14%	0.94	98.38%
PFUnA	84.46%	1.05	97.71%
PFDoA	83.50%	1.00	101.70%
PFTriA	78.03%	0.93	99.72%
PFTeA	76.57%	0.92	101.48%
PFPrS	96.93%	0.89	100.48%
PFBS	88.66%	1.05	99.09%
PFPeS	82.02%	0.94	99.82%
L-PFHxS	81.53%	0.92	100.84%
B-PFHxS	88.96%	1.03	101.05%
PFHpS	91.63%	1.07	97.99%
L-PFOS	90.58%	1.06	101.90%
B-PFOS	95.48%	1.24	98.87%
PFNS	79.96%	0.91	100.49%
PFDS	73.63%	0.86	98.79%
4:2 FTS	90.87%	1.09	99.66%
6:2 FTS	129.57%	1.64	101.06%
8:2 FTS	86.51%	0.92	101.91%
MeFOSAA	87.25%	0.99	100.33%
EtFOSAA	88.84%	1.10	101.42%
PFBSA	81.19%	3.14	100.66%
PFHxSA	76.39%	2.86	101.94%
PFOSA	85.52%	0.85	102.04%
3:3 FTCA	71.21%	0.93	99.30%
5:3 FTCA	78.43%	0.96	99.74%
7:3 FTCA	54.13%	0.70	98.33%
PFECHS	97.63%	1.17	102.97%
NFDHA	84.95%	1.00	99.18%
PFEESA	84.34%	1.01	98.78%
PFMPA	93.89%	1.07	99.36%
PFMBA	89.65%	1.05	100.67%
HFPO-DA	85.88%	1.30	99.79%
ADONA	84.81%	0.98	100.29%
9Cl-PF3ONS	79.36%	0.97	97.15%
11Cl-PF3OUdS	67.56%	0.85	97.86%

**Table 7:** TDCA interference study.

TDCA Concentration	L-PFOS (%Diff)	B-PFOS (%Diff)	PFNA (%Diff)
498.3 ng L^−1^	−6%	2%	10%
4.983 ng mL^−1^	−2%	13%	7%
49.83 ng mL^−1^	−4%	13%	4%
498.3 ng mL^−1^	−1%	−4%	3%
4.983 mg L^−1^	98% *	97% *	−3%
49.83 mg L^−1^	900%	892% *	−6%
498.3 mg L^−1^	**	**	**

* Linear and branched could not be distinguished. Total is integrated. ** Quantitation not possible due to high TDCA background.

**Table 8:** Maximum dilutions. A 500 ng mL^−1^ spiked matrix sample was prepared and then diluted 10,000× via either a four iterations of a 10× serial dilution, or an individual (single-step) 10,000× dilution.

	Serial Dilution %Error	Individual Dilution %Error
PFBA	3%	7%
PFPeA	−7%	1%
PFHxA	−6%	7%
PFHpA	−5%	0%
L-PFOA	1%	6%
B-PFOA	0%	0%
PFNA	−2%	−4%
PFDA	6%	0%
PFUnA	2%	−5%
PFDoA	4%	11%
PFTriA	−3%	−3%
PFTeA	1%	−8%
PFPrS	−10%	−5%
PFBS	3%	−3%
PFPeS	−7%	−7%
L-PFHxS	5%	6%
B-PFHxS	−8%	−11%
PFHpS	−4%	3%
L-PFOS	−4%	4%
B-PFOS	−10%	−14%
PFNS	1%	5%
PFDS	7%	5%
4:2 FTS	5%	0%
6:2 FTS	−7%	−3%
8:2 FTS	2%	−5%
MeFOSAA	−5%	8%
EtFOSAA	0%	−7%
PFBSA	−2%	−3%
PFHxSA	2%	−8%
PFOSA	−1%	−8%
3:3 FTCA	5%	7%
5:3 FTCA	4%	−6%
7:3 FTCA	−4%	7%
PFECHS	−6%	4%
NFDHA	8%	10%
PFEESA	11%	1%
PFMPA	−3%	−11%
PFMBA	4%	2%
HFPO-DA	−1%	−8%
ADONA	−3%	1%
9Cl-PF3ONS	5%	−3%
11Cl-PF3OUdS	−1%	8%

**Table 9:** Stability. Stability is shown as the % of analyte degraded over freeze/thaw cycles as compared to the control (never frozen), or 20 month stability as compared to the initial measurement of analyte in the sample. Analytes not present in the sample have their cells intentionally left blank (grey cell) and 20 month stability samples did not contain all analytes.

	%Degraded				
	Freeze/Thaw Cycles				Long-Term Stability
	2	3	4	5	20 Months
PFBA	3.5%	1.7%	2.8%	1.5%	−4.7%
PFPeA	−2.7%	−3.2%	−5.6%	−0.1%	−2.3%
PFHxA	0.2%	1.6%	4.0%	0.0%	2.9%
PFHpA	−4.2%	0.2%	−4.4%	−5.7%	−2.9%
L-PFOA	2.1%	−0.9%	7.2%	3.0%	9.4%
B-PFOA	0.0%	0.0%	0.0%	0.0%	0.0%
PFNA	−1.3%	−2.3%	−0.3%	−2.9%	−2.4%
PFDA	−0.1%	2.5%	2.2%	1.0%	4.8%
PFUnA	0.0%	1.1%	−1.0%	1.4%	1.6%
PFDoA	0.7%	4.9%	2.3%	0.0%	2.6%
PFTriA	4.1%	−1.1%	−3.2%	−2.8%	5.0%
PFTeA	−0.6%	3.1%	−0.9%	−2.3%	1.5%
PFPrS	−3.4%	−1.3%	2.8%	3.0%	
PFBS	1.2%	−0.3%	4.1%	2.3%	−8.0%
PFPeS	−6.0%	−1.1%	−7.6%	−3.7%	11.8%
L-PFHxS	−0.5%	4.2%	1.2%	−0.4%	−1.9%
B-PFHxS	−1.5%	4.3%	6.8%	0.6%	11.3%
PFHpS	4.5%	1.6%	4.3%	0.2%	5.8%
L-PFOS	2.7%	3.1%	0.1%	4.8%	−11.1%
B-PFOS	2.5%	1.3%	−3.9%	0.1%	8.1%
PFNS	1.5%	0.3%	−3.8%	−2.8%	4.2%
PFDS	0.5%	1.0%	0.3%	0.6%	4.8%
4:2 FTS	1.5%	1.8%	4.1%	−2.4%	4.9%
6:2 FTS	−0.4%	2.5%	1.9%	−2.1%	4.0%
8:2 FTS	−0.3%	3.3%	1.8%	−0.9%	9.5%
MeFOSAA	−2.3%	−3.7%	0.8%	−5.2%	4.8%
EtFOSAA	1.6%	5.0%	4.9%	0.9%	−6.2%
PFBSA	−1.5%	−1.3%	0.6%	−2.0%	
PFHxSA	3.3%	0.1%	−1.7%	−2.0%	
PFOSA	0.7%	3.3%	0.8%	−0.4%	9.0%
3:3 FTCA	3.2%	−0.5%	1.7%	3.3%	
5:3 FTCA	3.4%	−3.4%	−4.2%	−3.4%	
7:3 FTCA	7.3%	0.0%	1.8%	1.4%	
PFECHS	3.2%	−4.8%	−3.7%	2.3%	
NFDHA	1.2%	6.9%	4.1%	0.1%	
PFEESA	−4.1%	−5.1%	−5.7%	−5.4%	
PFMPA	−0.4%	1.7%	4.9%	1.5%	
PFMBA	4.3%	−1.1%	6.4%	2.5%	
HFPO-DA	−0.7%	−0.2%	0.6%	−3.3%	
ADONA	7.5%	1.8%	3.5%	2.3%	
9Cl-PF3ONS	−2.1%	−3.5%	−4.4%	3.2%	
11Cl-PF3OUdS	−4.6%	5.6%	−3.2%	−1.7%	

## Data Availability

The original contributions presented in this study are included in the article. Further inquiries can be directed to the corresponding author.
